# Host-Directed Antiviral Strategies Against Influenza Viruses: Host Targets, Multi-Omics Approaches and AI-Assisted Discovery

**DOI:** 10.3390/vetsci13070626

**Published:** 2026-06-27

**Authors:** Xianfeng Hui, Shihuan Ding, Shuoxiang Gao, Shuochen Xu, Tiesuo Zhao, Xiaowei Tian, Hui Wang

**Affiliations:** 1 Department of Immunology, School of Basic Medical Sciences, Henan Medical University, Xinxiang 453003, China; 50240206012@stu.xxmu.edu.cn (S.D.); 50250206064@stu.xxmu.edu.cn (S.G.); 20251450217@stu.xxmu.edu.cn (S.X.); 131009@hamu.edu.cn (T.Z.); 2 Henan Key Laboratory of Immunology and Targeted Drug, Henan Medical University, Xinxiang 453003, China; 3 Xinxiang Engineering Technology Research Center of Immune Checkpoint Drug for Liver-Intestinal Tumors, Henan Medical University, Xinxiang 453003, China; 4 Henan Collaborative Innovation Center of Molecular Diagnosis and Laboratory Medicine, School of Medical Technology, Henan Medical University, Xinxiang 453003, China

**Keywords:** influenza virus, host-directed antiviral strategies, multi-omics integration, artificial intelligence, host–virus interactions

## Abstract

Influenza viruses continue to pose a significant threat to both animal and public health due to their rapid evolution and the frequent emergence of antiviral resistance. Host-directed antiviral (HDA) strategies, which target host factors essential for viral replication, may represent an alternative to conventional virus-targeting approaches. However, the identification of reliable and therapeutically actionable host targets remains a major challenge, primarily due to the complexity and context dependency of host–virus interactions. Recent advancements in multi-omics technologies, including functional genomics, transcriptomics, and proteomics, have facilitated the systematic characterization of host factors involved in influenza virus infection. These methodologies have unveiled intricate regulatory networks that govern viral replication and host immune responses. Nonetheless, translating large-scale datasets into biologically meaningful targets necessitates robust integrative frameworks. In this context, artificial intelligence (AI) and machine learning methods offer powerful tools for data integration, target prioritization, and predictive modeling. In this Review, we summarize current insights into host factors that regulate influenza virus infection and discuss how multi-omics and AI-driven approaches are expediting host target discovery. Furthermore, we highlight the potential of these strategies to enhance antiviral development while addressing key challenges related to specificity, safety, and translational application. Collectively, these advancements lay a foundation that may support the rational design of next-generation host-directed antivirals.

## Simple Summary

Influenza viruses remain a major threat to animal and public health because of their rapid evolution and increasing antiviral resistance. Host-directed antiviral strategies, which target host factors required for viral replication, provide a promising alternative to traditional virus-targeting therapies. Recent advances in multi-omics technologies and artificial intelligence (AI) have improved the identification of host factors involved in influenza infection and accelerated the discovery of new antiviral targets. This review summarizes current knowledge of host factors regulating influenza virus infection and highlights how multi-omics and AI-driven approaches may support the development of next-generation antiviral therapies for both animals and humans.

## 1. Introduction

Influenza viruses continue to circulate and evolve within the One Health ecosystem, which includes humans, swine, poultry, and wild birds. Swine and avian species serve as significant reservoir hosts for influenza viruses, providing ecological environments that facilitate viral persistence, interspecies transmission, and genetic reassortment [1]. Consequently, controlling influenza is not only a public health concern but also a major challenge for animal health, food security, and the sustainable development of livestock production systems.

Due to their segmented genomes and high mutation rates, influenza viruses undergo continuous antigenic drift and can generate novel reassortant strains through the exchange of gene segments between viruses originating from different host species [2,3]. Such evolutionary dynamics have repeatedly contributed to the emergence of viruses with epidemic or pandemic potential [1,4]. In domestic animals such as swine and poultry, the sustained circulation of influenza viruses not only results in substantial economic losses but also increases the risk of zoonotic transmission and the emergence of novel influenza variants with public health significance [5,6]. Therefore, understanding influenza virus–host interactions across multiple host species has become increasingly important within the One Health framework.

Current anti-influenza strategies primarily target viral proteins, such as neuraminidase and the M2 ion channel [7]. However, the high mutation frequency of influenza viruses has led to the emergence of antiviral resistance, while extensive genetic and antigenic diversity among viral strains limits the long-term effectiveness of virus-directed therapies [7,8]. These limitations have stimulated growing interest in the development of antiviral strategies that are less susceptible to viral evolution.

Host-directed antiviral (HDA) strategies may represent an alternative approach by targeting host factors required for viral replication, although their application remains limited by safety and translational constraints. In parallel, increasing attention has been directed toward the identification of conserved host pathways that may be associated with antiviral effects across multiple influenza virus subtypes and, potentially, across different host species. In this review, the term “broad-spectrum antiviral strategy” is used primarily to describe interventions targeting conserved host mechanisms with activity against multiple influenza virus subtypes. Where appropriate, the terms “cross-subtype,” “cross-virus,” and “cross-host” are used to distinguish activity across influenza subtypes, different viral species, and different host organisms, respectively. Despite their potential advantages, host-targeted approaches face several challenges. Host–virus interactions are highly context-dependent and can vary substantially among cell types, tissues, and host species. Moreover, many host factors participate in essential cellular processes, raising concerns regarding safety, tissue specificity, and therapeutic windows.

Recent advances in multi-omics technologies have provided unprecedented opportunities to systematically characterize host regulatory networks during influenza virus infection. Functional genomics, transcriptomics, proteomics, and interactomics collectively enable the identification of host factors involved in viral replication, immune regulation, and host adaptation [9]. However, the scale, complexity, and heterogeneity of these datasets present significant challenges for conventional analytical approaches. In this context, artificial intelligence (AI) and machine learning methods have emerged as powerful tools for data integration, network analysis, and target prioritization [10]. By combining multi-omics evidence with computational predictions, AI-assisted approaches may help prioritize biologically relevant host factors, although their outputs require careful experimental validation.

This review summarizes current studies on influenza host targets across multiple host species, with particular emphasis on the integration of multi-omics technologies and AI-assisted approaches. Special attention is given to conserved host factors that have been proposed as candidates for cross-subtype influenza control and their relevance in humans, swine, and poultry. Additionally, we discuss the challenges associated with translating host-target discoveries across species, the limitations of AI-assisted target prioritization, and the opportunities for developing host-targeted antiviral strategies within a One Health framework.

This article is a narrative review. Relevant literature was identified through searches of PubMed, Web of Science, and Scopus using combinations of keywords including “influenza virus”, “host factors”, “host-directed antiviral”, “multi-omics”, “functional genomics”, “transcriptomics”, “proteomics”, “interactomics”, “artificial intelligence”, “machine learning”, and “One Health”. Priority was given to peer-reviewed publications with particular emphasis on recent studies published within the last five years, while seminal studies were included where necessary to provide historical and mechanistic context. No formal language restrictions were applied during literature selection; however, the vast majority of the included studies were published in English.

## 2. Host Factors in Influenza Virus Infection

Influenza virus replication relies extensively on host cellular machinery and structural components, making infection a dynamic process shaped by continuous interactions between viral factors and host regulatory networks. From viral entry and genome replication to particle assembly and release, each stage of the viral life cycle depends on host membrane trafficking systems, nucleocytoplasmic transport pathways, and diverse metabolic and signaling processes. At the same time, host cells detect viral invasion through innate immune sensing mechanisms and initiate antiviral defenses. However, influenza viruses have evolved multiple strategies to counteract or evade these responses. Consequently, host factors involved in influenza infection can generally be classified into two categories: proviral factors that facilitate viral replication and restriction factors that suppress viral propagation. The functions and relative importance of these factors vary according to cell type, stage of infection, and host species. Notably, many host pathways involved in influenza virus replication and immune regulation exhibit varying degrees of conservation across species, providing opportunities for the identification of host-directed antiviral targets with potential cross-subtype or cross-species applicability. At the same time, the essential physiological roles of many host factors raise important concerns regarding safety, selectivity, and therapeutic windows. Therefore, a systematic understanding of the functional categories of host factors and their roles throughout the influenza virus life cycle is fundamental to elucidating viral pathogenesis and advancing host-targeted antiviral strategies.

### 2.1. Host Factors Related to the Viral Life Cycle

Influenza virus replication depends on multiple fundamental biological processes within host cells, with distinct yet interconnected host factors contributing to different stages of the viral life cycle. According to their functions, these host factors can be broadly grouped into those involved in viral entry, nuclear replication, and virion assembly and release.

During viral entry, influenza viruses initially attach to sialic acid receptors on the host cell surface, such as α-2,6-linked sialic acid receptors that predominate in human airway epithelial cells, through interactions mediated by hemagglutinin (HA) [11]. The virus is subsequently internalized via clathrin-mediated endocytosis [12]. Following uptake, progressive acidification of endosomes triggers fusion between the viral envelope and the endosomal membrane [13]. This process depends on endosomal acidification machinery, including the v-ATPase complex [13], as well as ion channel activity. Simultaneously, proton influx mediated by the viral M2 protein promotes uncoating and facilitates the release of viral ribonucleoprotein complexes (vRNPs) into the cytoplasm [14]. Host regulators of endosomal trafficking, particularly Rab5 and Rab7, are required for endosome maturation and efficient viral genome release [15]. Notably, these pathways are highly conserved across mammalian and avian hosts, highlighting their potential relevance as host-directed antiviral targets.

During the replication stage, influenza viruses exhibit a unique dependence on the host nuclear transport machinery because viral transcription and genome replication occur within the nucleus. Nuclear import of vRNPs is primarily mediated by the importin-α/β transport system [16], including importin-α7 [16], which recognizes viral nuclear localization signals and facilitates translocation into the nucleus. Viral mRNA synthesis relies on the well-characterized cap-snatching mechanism [17], whereby the viral polymerase complex utilizes the 5′ cap structures of host RNA polymerase II transcripts as primers for transcription initiation. In this process, the PB2 subunit specifically binds host-derived capped RNA fragments [18]. Host transcriptional activity and chromatin organization also influence viral replication efficiency, and RNA polymerase II activity has been shown to correlate closely with viral transcriptional output [19].

Among the host factors involved in viral replication, members of the ANP32 protein family represent particularly important regulators of influenza polymerase activity and host adaptation [20]. ANP32 proteins are broadly conserved among humans, swine, and avian species; however, their ability to support viral polymerase function differs substantially between hosts. For example, avian ANP32A contains a species-specific insertion that efficiently supports avian influenza polymerases, whereas mammalian ANP32A and ANP32B exhibit distinct levels of support depending on the degree of viral adaptation [20]. These differences are now recognized as important determinants of host range and cross-species transmission [20,21]. Similarly, the importin-α/β-mediated nuclear transport pathway operates in human, porcine, and avian cells [16], indicating that nucleocytoplasmic transport represents a conserved host dependency pathway that may offer opportunities for the development of host-directed antiviral interventions across multiple host systems.

**Figure 1:**
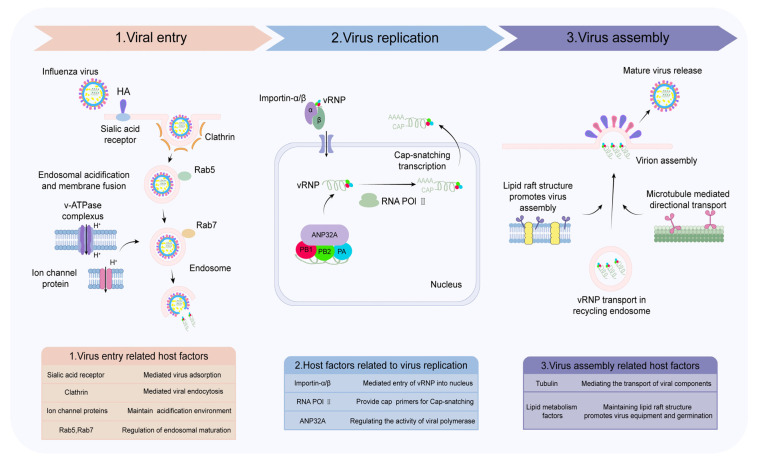
Host factors involved in the influenza virus life cycle.

During virion assembly and release, newly synthesized viral proteins and genomic components are transported to the plasma membrane through host vesicular trafficking pathways [22]. This process involves Golgi-associated transport mechanisms and cytoskeletal networks, particularly microtubules, which facilitate the coordinated trafficking of viral proteins and vRNPs to assembly sites [23]. Host lipid metabolism also contributes substantially to virion formation. For example, cholesterol- and sphingolipid-enriched lipid rafts provide specialized membrane microdomains that support viral assembly and budding [24]. Mature virions are ultimately released from infected cells through a budding process, during which neuraminidase (NA) cleaves terminal sialic acid residues to prevent virion aggregation and promote the efficient release of progeny viruses [25,26] (Figure 1).

Influenza virus replication relies on multiple host factors that regulate distinct stages of the viral life cycle. During viral entry, the virus binds to sialic acid receptors on the host cell surface and is internalized through endocytosis. Endosomal acidification mediated by the v-ATPase complex and endosomal trafficking regulators such as Rab5 and Rab7 facilitates viral membrane fusion, uncoating, and release of viral ribonucleoprotein complexes (vRNPs). During nuclear replication, vRNPs are transported into the nucleus through the importin-α/β nuclear transport system, where viral transcription and genome replication occur. Host factors such as ANP32 proteins support influenza polymerase activity and contribute to host adaptation. During virion assembly and release, newly synthesized viral components are transported through vesicular trafficking pathways and assembled at cholesterol-rich lipid rafts on the plasma membrane. Host lipid metabolism and cytoskeletal networks play important roles in this process, while neuraminidase-mediated budding enables the release of progeny virions. Collectively, these host pathways represent critical determinants of influenza virus replication and potential targets for host-directed antiviral strategies.

Collectively, each stage of the influenza virus life cycle depends on host cellular pathways that regulate intracellular trafficking, nuclear transport, metabolism, and membrane dynamics. These host factors not only influence viral replication efficiency but also contribute to host adaptation and cross-species transmission. Importantly, many of these pathways exhibit varying degrees of conservation among humans, swine, and avian species, making them attractive candidates for host-directed antiviral intervention. Therefore, systematic characterization of host dependency factors across different stages of infection provides an essential foundation for the identification and prioritization of antiviral targets.

### 2.2. Host Antiviral Immune Regulation

The innate immune system constitutes the first line of host defense during the early stages of influenza virus infection. Following viral entry, viral RNA is detected by pattern-recognition receptors (PRRs), among which the RIG-I-like receptor pathway plays a particularly important role [27]. Recognition of viral RNA by RIG-I triggers MAVS-dependent signaling [28], leading to the activation of downstream kinases and transcription factors that induce the production of type I interferons and pro-inflammatory cytokines [29]. The magnitude and duration of this response are regulated by multiple host factors, including ubiquitin-modifying enzymes, protein kinases, and transcriptional regulators, which collectively shape antiviral immunity.

The interferon signaling pathway represents one of the major host mechanisms restricting influenza virus replication. Binding of type I interferons to their cognate receptors activates the JAK–STAT pathway and stimulates the expression of numerous interferon-stimulated genes (ISGs) [30], which inhibit viral replication through diverse mechanisms. Representative examples include IFITM proteins, which restrict viral membrane fusion [31], and Mx proteins, which interfere with viral ribonucleoprotein function and replication [32]. Additional ISGs contribute to antiviral defense by regulating RNA degradation, protein translation, intracellular trafficking, and other cellular processes, thereby establishing a multi-layered antiviral network [33].

However, influenza viruses have evolved multiple mechanisms to counteract host innate immunity. Viral proteins can disrupt key components of the RIG-I–MAVS signaling axis or suppress interferon production and downstream signaling, thereby attenuating antiviral responses. Importantly, although many innate immune pathways are conserved among humans, swine, and avian species, substantial differences exist in immune regulation, signaling intensity, and antiviral effector repertoires. These species-specific variations influence viral adaptation, pathogenicity, and transmission dynamics and should be considered when evaluating host-directed antiviral targets across different host systems (Table 1).

### 2.3. Host-Dependent Factors and Restriction Factors

Based on their roles in viral replication, host factors are typically classified into proviral factors, which promote viral replication, and restriction factors, which inhibit viral amplification. Proviral factors provide the cellular environment and molecular support required for viral replication, including host components involved in nuclear transport, protein translation, membrane trafficking, and energy metabolism. Taking influenza viruses as an example, members of the importin-α family mediate the nuclear import of viral vRNP complexes [16]. In addition, ANP32A supports the activity of viral polymerase complexes and plays a particularly important role in the adaptation of avian influenza viruses to mammalian hosts [20].

Additionally, viral replication relies on the host translation machinery and lipid metabolism pathways to support viral protein synthesis and virion assembly [34]. In contrast, restriction factors primarily suppress viral replication through direct or indirect mechanisms. For example, IFITM3 can restrict viral entry by interfering with viral membrane fusion [31]; MxA (Mx1) can inhibit viral replication by disrupting the function of viral ribonucleoprotein complexes [35]; and PKR suppresses protein translation upon recognizing viral RNA, thereby limiting viral amplification [36]. Furthermore, certain host nucleases and ubiquitination-related factors are involved in viral RNA degradation and the regulation of antiviral signaling [37]. It should be noted that the functions of host factors are not strictly fixed. Some host molecules may exhibit dual roles at different stages of infection or in different cellular contexts. For example, autophagy-related pathways may promote the transport and replication of viral components under certain conditions, whereas under other conditions, they may facilitate viral clearance and antigen presentation [38]. Similarly, factors associated with cellular stress responses may either be exploited by viruses to maintain a favorable replication environment or participate in host antiviral defense [39].

**Figure 2:**
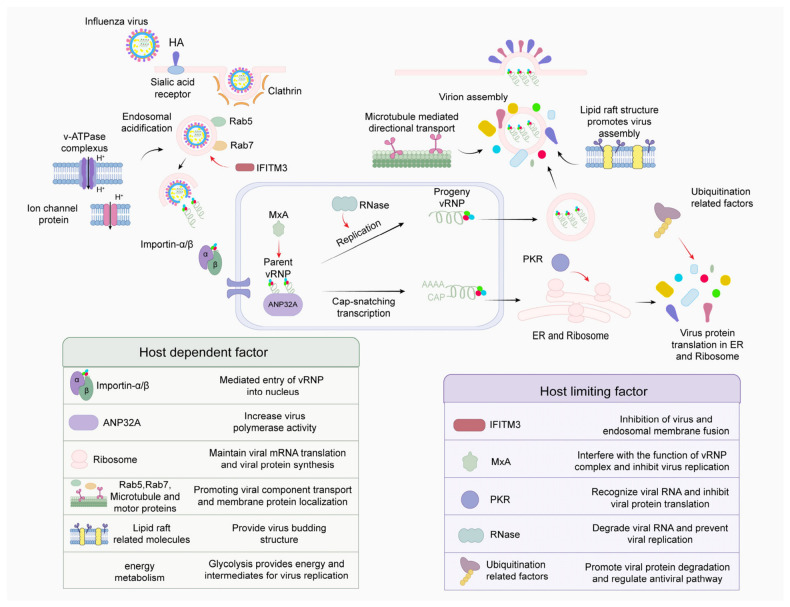
Host-dependent and restriction factors during influenza virus infection.

Furthermore, functional redundancy and pathway compensation are widespread within host cells [40]. When a single host factor is inhibited, viruses may maintain their replication capacity by utilizing alternative pathways. This phenomenon may limit the effectiveness of single-target interventions and increase the complexity of designing host-targeted antiviral strategies. It is noteworthy that some host dependency factors and restriction factors exhibit high levels of conservation across humans, pigs, and birds. For instance, the nuclear transport system, endocytic trafficking pathways, and interferon-induced antiviral responses are extensively involved in influenza virus infection across different hosts [41,42]. This conservation provides an important foundation for the identification of broad-spectrum antiviral targets. However, these pathways often participate in essential physiological functions, and excessive interference may result in cytotoxicity or immune imbalance. Therefore, the screening of host targets should comprehensively consider antiviral efficacy, host safety, and functional differences among species (Figure 2).

Overall, influenza virus replication depends on a complex host regulatory network comprising both host dependency factors that facilitate viral replication and antiviral factors that restrict viral amplification. These host factors undergo dynamic changes across different stages of infection, cell types, and host species, and their interactions collectively influence viral replication efficiency and host adaptability. Systematic analysis of host-factor networks and their dynamic balance provides an important basis for the development of host-targeted antiviral strategies and broad-spectrum antiviral targets (Table 1).

Key host factors facilitating or restricting viral replication are depicted across entry, nuclear replication, and assembly stages. Several factors (e.g., ubiquitination-related proteins, Importin-α/β, RNase) exert dual pro- and antiviral functions depending on context. Energy metabolism, particularly glycolysis, supports viral biosynthesis, while restriction factors, including IFITM3, Mxα, and PKR, antagonize critical steps of the viral life cycle.

## 3. Application of Multi-Omics Technologies in Host Target Discovery

With the advancement of high-throughput sequencing and systems biology technologies, multi-omics approaches have become a key tool for elucidating the interactions between influenza viruses and their hosts. Omics data from various levels provide complementary information across multiple dimensions—from gene function and transcriptional regulation to protein interactions—enabling the identification of host factors to shift from single-dimensional studies to systematic analysis. By integrating data from multiple sources, researchers can not only map host regulatory networks during viral infection more comprehensively but also establish a basis for screening targets with biological significance and potential application value.

### 3.1. Functional Genomics

Functional genomics is a key approach for the systematic identification of host factors associated with influenza virus infection. Early studies primarily relied on RNA interference (RNAi) technology, using siRNA or shRNA to silence specific genes and evaluate the effects of host gene depletion on viral replication [9]. RNAi-based screening has identified numerous host factors involved in the influenza virus life cycle, including the COPI complex, which participates in viral endocytosis and intracellular trafficking, NXF1, which regulates nuclear transport, and ATP6V0D1, which is associated with viral RNA replication [9]. These studies established an important foundation for subsequent host-target discovery efforts.

In recent years, the development of CRISPR/Cas9 technology has substantially improved the accuracy and throughput of functional screening. Genome-wide CRISPR-based knockout screens enable systematic investigation of host gene functions and facilitate the identification of host dependency factors that are critical for viral replication. For example, several CRISPR screening studies have demonstrated that disruption of the sialic acid biosynthesis genes *SLC35A1* and *CMAS* markedly reduces cellular susceptibility to influenza virus infection, highlighting their importance in viral entry [43]. In addition, CRISPR screens have identified *ANP32A* and *ANP32B* as key regulators of influenza virus polymerase activity [44]. Beyond gene knockout approaches, CRISPR interference (CRISPRi) and CRISPR activation (CRISPRa) technologies allow targeted suppression or enhancement of gene expression, enabling more flexible investigation of host-factor functions across different expression levels [45]. Compared with conventional RNAi approaches, CRISPR screening provides greater targeting specificity and fewer off-target effects, making it a more reliable platform for defining host gene function [46].

To date, most CRISPR screening studies have been conducted in human-derived cell lines. However, functional genomics studies using porcine and avian cells have increased in recent years. Cross-species comparative screening can facilitate the identification of conserved host factors involved in viral replication across different hosts, thereby improving the efficiency of discovering broadly applicable antiviral targets. For example, differences in the ability of the ANP32 protein family to support influenza virus polymerase activity have been observed between human and avian cells, and these differences are thought to contribute to host adaptation and cross-species transmission [47]. Consequently, integrating functional genomics data from multiple host species may help identify conserved host targets that play important roles across different biological systems and provide a foundation for the development of host-targeted antiviral strategies.

Functional genomics screening is often regarded as the starting point of host-target discovery. However, the large number of candidate genes identified through RNAi or CRISPR screening requires further prioritization through integration with transcriptomic and proteomic datasets. For example, when a candidate gene not only influences viral replication in CRISPR screens but also exhibits significant expression changes during infection and occupies a central position within host regulatory networks, confidence in its potential as an antiviral target is substantially strengthened. Therefore, cross-validation of functional screening results with complementary omics datasets has become an important strategy in contemporary host-target research (Table 2).

### 3.2. Transcriptomics

Transcriptomic analysis provides important insights into the dynamic host response to influenza virus infection. Using RNA sequencing (RNA-seq), researchers can systematically compare host gene expression profiles before and after infection, thereby identifying key pathways involved in antiviral defense or exploited by the virus. For example, in influenza virus-infected lung epithelial cells, multiple interferon-stimulated genes (ISGs), including *IFITM3*, *ISG15*, and members of the *OAS* family, are typically upregulated, reflecting rapid activation of innate immune responses [48]. At the same time, inflammatory cytokines and chemokines, such as IL-6 and CXCL10, are often highly expressed and are closely associated with disease pathogenesis [49]. Traditional bulk RNA-seq primarily captures the average transcriptional profile of an entire cell population and is therefore well-suited for analyzing global transcriptional changes following infection. For example, studies have shown that infection with highly pathogenic H5N1 viruses induces stronger expression of inflammation-related genes, whereas low-pathogenicity strains generally elicit more moderate transcriptional responses. Such findings provide insights into differences in pathogenic mechanisms among influenza virus strains. In recent years, single-cell RNA sequencing (scRNA-seq) has further revealed the cellular heterogeneity associated with viral infection [50]. For instance, within influenza virus-infected lung tissue, individual cells can differ substantially in viral burden and immune activation; some cells exhibit robust interferon responses, whereas others are more permissive to viral replication [50]. Single-cell analyses therefore facilitate the identification of host factors associated with specific cell types and potentially susceptible cellular populations.

Furthermore, transcriptomic analysis has been widely applied in studies of swine and avian influenza to investigate host immune responses and mechanisms of viral adaptation. For example, although human, swine, and avian cells all activate interferon-related pathways following influenza virus infection, differences have been observed in the expression patterns of certain inflammatory mediators and metabolism-related genes [51,52]. Such cross-species transcriptomic comparisons help identify genes that exhibit conserved response patterns across different hosts and provide a foundation for the discovery of broadly applicable antiviral targets. However, transcriptomic data alone are insufficient to determine the functional importance of candidate genes. Many differentially expressed genes do not directly contribute to viral replication, whereas some critical host factors show little or no transcriptional change during infection. Therefore, transcriptomic findings are often interpreted in conjunction with functional genomics and proteomics data. For example, a host factor that exhibits infection-associated expression changes and is independently validated in CRISPR screening is more likely to represent a biologically relevant antiviral target.

### 3.3. Proteomics and Interactomics

Proteomics and interactomics reveal host–virus interactions at the protein level and serve as a crucial link between changes in gene expression and biological function. Compared with transcriptomics, proteomics can more directly reflect alterations in the functional state of host cells following viral infection. Mass spectrometry (MS)-based analytical techniques enable the systematic detection of changes in host protein abundance and post-translational modifications, such as phosphorylation and ubiquitination, during infection [53]. These modifications are often closely associated with the regulation of cellular signaling pathways.

In influenza virus research, proteomics has been widely used to analyze alterations in host signaling networks following infection. For example, studies have shown that influenza virus infection can significantly affect the phosphorylation status of proteins involved in signaling pathways such as MAPK and PI3K–Akt, and these changes are closely linked to viral replication and host inflammatory responses [9,54]. Furthermore, viral infection can also alter the expression of host metabolic and cytoskeletal proteins, thereby modifying the intracellular environment to facilitate viral replication [55].

Direct interactions between viral and host proteins constitute a crucial foundation for successful viral replication. Using techniques such as immunoprecipitation coupled with mass spectrometry (IP–MS), yeast two-hybrid assays, and proximity-labeling approaches (e.g., BioID and APEX), virus–host protein interaction networks can be systematically constructed. For example, the influenza virus NS1 protein interacts with host proteins such as TRIM25 and CPSF30 [56,57], thereby suppressing RIG-I-mediated interferon responses, whereas the viral polymerase subunit PB2 binds to the host protein ANP32A to maintain polymerase complex activity [20]. These interacting factors often play critical roles in viral replication or immune evasion and are therefore considered potential targets for intervention.

Meanwhile, cross-species interactomics studies have shown that some virus–host protein interactions are highly conserved across different host species, whereas others exhibit substantial host specificity. Comparative analysis of interaction networks in human-, porcine-, and avian-derived cells can facilitate the identification of host targets with broad-spectrum intervention potential.

Although multi-omics technologies provide valuable resources for host-target discovery, differences in data structure, experimental design, and temporal dynamics continue to create challenges for data integration and interpretation. These limitations have motivated the development of computational and AI-assisted approaches for multi-omics integration, which are discussed in the following section.

### 3.4. Multi-Omics Integration Drives Host Target Discovery

Single-omics studies often capture only limited aspects of the host response, whereas influenza virus replication is regulated through coordinated processes operating at multiple molecular levels. Consequently, multi-omics integration has emerged as an important strategy for host-target discovery.

A typical workflow begins with the identification of candidate host factors that influence viral replication through CRISPR- or RNAi-based screening [58]. Transcriptomic analyses are then used to characterize the expression dynamics of these genes during infection. Subsequently, proteomic and interactomic approaches are employed to investigate the associated signaling pathways and virus–host protein interactions. Finally, network analysis, machine learning, and experimental validation are integrated to prioritize and functionally verify candidate targets.

ANP32A provides a representative example of this process [20]. It was initially identified in functional screening studies as a host factor influencing influenza virus polymerase activity. Subsequent transcriptomic and proteomic analyses further demonstrated its involvement in the regulation of viral replication complexes and revealed substantial functional differences among cells derived from different host species. These findings ultimately established ANP32A as an important determinant of influenza virus cross-species adaptation [59]. Similarly, host factors such as *SLC35A1*, *CMAS*, members of the importin-α family, and several interferon-related factors have progressed through a comparable research pipeline, from initial screening to multi-omics validation [43].

By integrating functional genomics, transcriptomics, proteomics, and interactomics datasets, researchers can not only improve the accuracy of host-target identification but also uncover core regulatory nodes that are shared across different host species and influenza virus subtypes [60]. These conserved host factors may represent potential candidates for host-directed antiviral intervention and provide an important foundation for the development of broadly applicable antiviral strategies while also generating the data resources required for subsequent AI-assisted target prioritization.

Overall, multi-omics technologies have become a fundamental platform for host-target discovery. However, the rapid accumulation of CRISPR screening, RNA-seq, proteomic, and interactomic datasets has greatly increased both data volume and analytical complexity. Identifying host targets that possess antiviral potential while maintaining an acceptable safety profile remains a major challenge. As a result, artificial intelligence approaches—including network analysis, machine learning, and deep learning—are increasingly being incorporated into host-target research. By integrating diverse multi-omics datasets, these methods can prioritize candidate targets, predict their biological functions, and improve the efficiency of antiviral target discovery.

## 4. AI-Driven Target Integration and Screening

With the rapid development of multi-omics technologies, including CRISPR screening, transcriptomics, proteomics, and interactomics, vast amounts of high-dimensional, heterogeneous, and dynamically changing datasets have been generated during influenza virus infection. However, individual omics layers often capture only limited aspects of the host response, and discrepancies frequently exist among different data types. For example, transcriptional changes do not necessarily correlate with protein abundance, while post-translational modifications further increase the complexity of biological regulation. Consequently, systematically identifying key host factors that genuinely contribute to viral replication from complex multi-omics datasets has become a major challenge in host-target discovery.

The emergence of artificial intelligence (AI) and machine learning technologies has provided powerful tools to address this challenge. By integrating information derived from functional genomics, transcriptomics, proteomics, and interactomics, AI-based approaches can reconstruct host regulatory networks, identify key nodes associated with viral replication, and prioritize candidate host targets. In recent years, a variety of computational methods, including network analysis, machine learning, and deep learning, have increasingly been applied to the study of influenza virus host factors. These approaches have demonstrated considerable potential for the identification and prioritization of host-directed antiviral (HDA) targets, as well as host factors that may support the development of broadly applicable antiviral strategies.

### 4.1. Network Analysis Aids in Host Target Discovery

Host–virus interactions during influenza infection form complex regulatory networks involving viral proteins, host dependency factors, signaling pathways, and immune regulators. Network-based approaches have therefore become widely used for identifying host factors that occupy central positions within influenza replication networks. Network-based analytical approaches integrate information from protein–protein interactions, signaling pathways, gene co-expression patterns, and functional annotations to construct host regulatory networks and identify key nodes that play critical roles in viral replication [61].

In influenza virus research, virus–host protein interaction networks have been widely used to identify important host factors. For example, analysis of the influenza virus NS1 interaction network has revealed interactions with several host proteins, including TRIM25 and CPSF30 [56,57], thereby suppressing interferon production and promoting viral immune evasion. Similarly, the interaction between PB2 and ANP32A has been shown to be closely associated with viral polymerase activity and host adaptation [20]. Such analyses facilitate the identification of host factors occupying central regulatory positions during viral replication. Meanwhile, network-analysis approaches are commonly used to prioritize host factors identified from CRISPR screens, transcriptomic studies, and virus–host interaction datasets by identifying regulatory nodes that are repeatedly implicated across multiple datasets. For example, host pathways involved in nuclear transport, endosomal trafficking, and innate immune signaling have frequently emerged as central regulatory modules in influenza-associated host networks, which are often involved in multiple biological processes. Proteins associated with signaling pathways such as PI3K–Akt and MAPK have repeatedly been implicated in influenza virus replication [62]. Functional-module analysis, in contrast, helps identify groups of genes or proteins that undergo coordinated changes during infection, including modules associated with immune responses, nuclear transport, and metabolic regulation. Furthermore, network analysis can be used to compare regulatory differences among host species. For example, comparative analyses of host interaction networks in human and avian cells have shown that certain nodes involved in nuclear transport and innate immunity are highly conserved across species [41], whereas inflammatory regulatory pathways exhibit greater divergence. Such cross-species network comparisons facilitate the identification of conserved regulatory nodes that may play important roles in multiple hosts and therefore represent promising candidates for host-directed antiviral intervention.

In practice, network analysis is often performed after the integration of multi-omics datasets. Host dependency factors identified through CRISPR screening, differentially expressed genes identified by RNA-seq, differentially expressed proteins identified through proteomics, and virus–host interaction data can be incorporated into a unified network framework [58,63]. After integrating CRISPR-screening, transcriptomic, proteomic, and virus–host interaction datasets, network-based approaches can be used to identify host factors that are repeatedly implicated across multiple biological layers. Such factors are more likely to play biologically relevant roles in influenza replication and therefore represent higher-confidence host-target candidates. These nodes are generally considered to have greater biological relevance and are therefore prioritized as potential host targets. For example, one study constructed an influenza virus–host interaction network and found that multiple host proteins involved in RNA processing, signal transduction, and immune regulation formed highly interconnected functional modules that were closely associated with viral replication [64]. Similarly, another study integrated RNAi-screening results with protein-interaction networks and identified several key host factors involved in viral replication, including the COPI complex and v-ATPase [9]. Collectively, these findings demonstrate that network analysis can substantially improve the efficiency of host-target discovery and assist in identifying biologically meaningful regulatory nodes from large candidate gene sets.

### 4.2. Machine Learning-Assisted Host Factor Prediction

Machine learning approaches can identify host factors associated with influenza viral replication from complex multidimensional datasets. Compared with traditional statistical methods, machine learning can not only process large-scale datasets but also capture complex nonlinear relationships among biological variables. As a result, it has become an increasingly important tool for host-target discovery in recent years [65,66].

Machine-learning approaches have increasingly been applied to prioritize influenza host factors by integrating CRISPR-screening results, transcriptomic profiles, protein–protein interaction networks, and functional annotations [67]. Algorithms including Random Forest, Support Vector Machine (SVM), and XGBoost have been widely applied to prioritize candidate host factors and identify pathways associated with influenza replication [68]. For example, by integrating CRISPR screening results, RNA-seq datasets, and protein–protein interaction networks as model inputs, machine-learning approaches can identify host pathways associated with viral replication, including nuclear transport, RNA processing, and endocytic trafficking [69,70]. Machine learning has also been extensively applied to the prediction of influenza virus–host protein interactions. In these studies, features such as protein-sequence characteristics, domain composition, and functional annotations are extracted and incorporated into predictive models, including SVM, Random Forest, and XGBoost [71,72]. These approaches enable the identification of potential influenza virus–host interactions and facilitate the discovery of novel candidate host factors. Compared with experimental screening methods, computational prediction offers advantages in terms of cost efficiency, scalability, and coverage [72].

Machine-learning approaches have also been used to identify co-regulated host pathways and compare host responses across viral strains, infection stages, and host species. For example, clustering analyses can identify gene modules that exhibit coordinated changes during infection [73]. In addition, machine-learning approaches have also been applied to compare host responses across viral strains, infection stages, and host species, facilitating the identification of conserved regulatory pathways associated with influenza infection [74].

In the future, machine-learning models may further integrate transcriptomic, CRISPR-screening, and protein–protein interaction datasets derived from human, porcine, and avian cells to establish cross-species predictive frameworks. Such approaches may improve the identification and prioritization of conserved host factors with potential value for broadly applicable antiviral intervention strategies.

### 4.3. Deep Learning and Multi-Omics Integration

Deep learning methods offer unique advantages for analyzing high-dimensional, multi-layered, and complex biological datasets. Compared with traditional machine learning approaches, which often rely on manual feature engineering, deep learning models can automatically learn informative features from diverse omics datasets through multi-layer neural network architectures, thereby improving the identification of key host factors [75].

In recent years, multi-omics integration has emerged as an important strategy for host-target discovery [60]. A typical workflow involves CRISPR screening, transcriptomic analysis, proteomic profiling, virus–host interactome mapping, AI-assisted data integration, candidate-target prediction, and experimental validation [76]. Through this process, biological information from multiple molecular layers can be systematically integrated, thereby improving the reliability of host-target identification. Deep-learning approaches have increasingly been explored for integrating complex influenza-related datasets, particularly virus–host interaction networks and multi-omics datasets. For example, Graph Neural Networks (GNNs) have become one of the most widely used deep learning models for biological network analysis [77]. Network-based deep-learning models have been used to prioritize host proteins that occupy central positions in influenza virus–host interaction networks and may therefore represent potential antiviral targets. Consequently, GNN-based approaches have increasingly been applied to the prediction of influenza virus–host protein interactions and the identification of host dependency factors [77]. Deep-learning approaches have also been applied to integrate transcriptomic, proteomic, and interactomic datasets, enabling the identification of candidate host factors supported by evidence from multiple molecular layers. By simultaneously integrating transcriptomic, proteomic, and interaction-network information, these models can uncover potential regulatory relationships that are difficult to detect using a single omics layer alone. Such integrative frameworks may be particularly valuable for identifying conserved host pathways shared among human, swine, and avian influenza infections. Future deep learning-driven multi-omics integration approaches may enable the simultaneous analysis of datasets derived from different host species, facilitate the identification of conserved host regulatory networks, and support the development of broadly applicable host-directed antiviral strategies [78].

**Figure 3:**
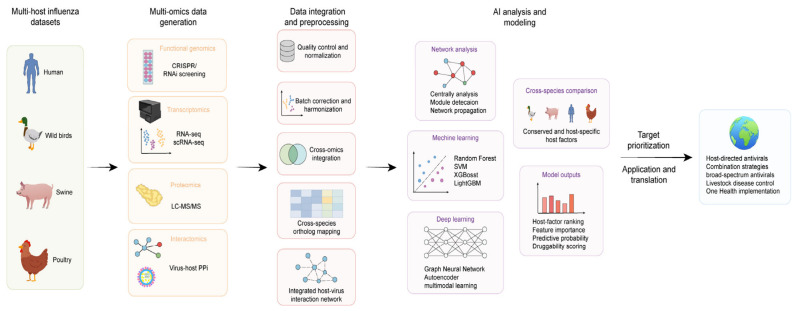
Multi-omics and artificial intelligence integration pipeline for host factor prioritization in influenza virus infection.

In livestock systems, the application of AI-assisted target prioritization faces additional challenges. Available multi-omics datasets from swine and poultry remain substantially smaller than those generated from human studies, resulting in data imbalance and reduced model robustness. Furthermore, differences in immune responses, genetic backgrounds, and production environments among livestock species may limit the transferability of predictive models developed using human-derived datasets. Therefore, future efforts should focus on establishing standardized cross-species datasets, improving model interpretability, and strengthening experimental validation to facilitate practical veterinary applications (Figure 3).

Despite these advances, relatively few AI-prioritized host factors have progressed to comprehensive mechanistic validation or in vivo evaluation in influenza models. Consequently, the major contribution of AI currently lies in improving target prioritization and hypothesis generation rather than replacing experimental screening and validation. Future studies should focus on integrating computational prediction with functional genomics and animal infection models to improve the translational value of AI-assisted host-target discovery.

This schematic outlines a systematic framework combining multi-host influenza datasets (human, swine, poultry, wild birds) with multi-omics profiling (functional genomics, transcriptomics, proteomics, and interactomics). Following data integration and cross-species ortholog mapping, AI-driven modeling—including network analysis, machine learning, and deep learning approaches—is applied to rank host factors based on feature importance, predictive probability, and druggability scoring. The prioritization pipeline ultimately supports host-directed antiviral development, broad-spectrum strategies, and One Health-oriented interventions for livestock and human health.

## 5. Host-Directed Antiviral (HDA) Strategies

Based on a clear understanding of host dependency factors and their regulatory networks, antiviral strategies that target host pathways rather than viral components have attracted increasing attention. Unlike conventional antiviral approaches that directly target viral proteins, host-directed antiviral (HDA) strategies may reduce the likelihood of antiviral resistance by interfering with host processes required for viral replication. In addition, because some host pathways are utilized by multiple influenza virus subtypes and, in certain cases, by different RNA viruses, host-targeted approaches may offer broader applicability than virus-specific therapies. However, many host targets also participate in essential cellular functions, raising concerns regarding toxicity, tissue specificity, and therapeutic windows. Therefore, the development of HDA strategies requires careful balancing of antiviral efficacy with host safety.

### 5.1. Targeting Key Host Pathways

Intervening in key host pathways that are essential for viral replication is a major focus of host-targeted antiviral research. During infection, influenza viruses are highly dependent on fundamental host cellular functions, including endocytic trafficking, nucleocytoplasmic transport, protein translation, and lipid metabolism [79]. Therefore, pharmacological interventions targeting these host pathways hold promise for inhibiting viral replication at multiple stages.

During the viral entry stage, endosomal acidification is essential for fusion between the viral envelope and the endosomal membrane. Several compounds capable of modulating endosomal pH have demonstrated anti-influenza activity. For example, Bafilomycin A1 has been widely used as a mechanistic research tool to demonstrate the importance of v-ATPase-mediated endosomal acidification during influenza virus entry [80]. However, its substantial cytotoxicity limits its direct therapeutic applicability; therefore, it primarily serves as proof-of-concept evidence supporting endosomal acidification as a potential host-targeted antiviral pathway. Similarly, chloroquine and other weakly basic compounds have been widely used as experimental tools to investigate the pH dependence of influenza virus entry [81]. Although antiviral activity has been observed in certain in vitro systems, translational evidence remains limited, and these compounds have not demonstrated consistent efficacy as clinically applicable host-directed anti-influenza therapies. Influenza virus replication also relies on the host nuclear transport system. The nuclear import of viral vRNPs is primarily mediated by importin-α/β [16], whereas the export of newly synthesized vRNPs from the nucleus involves nuclear export pathways such as CRM1/exportin 1 [82]. Similarly, Leptomycin B has been extensively used as an experimental inhibitor to investigate the role of CRM1/exportin 1 in vRNP nuclear export [64]. Owing to its toxicity profile, it is generally regarded as a mechanistic tool compound rather than a clinically applicable antiviral agent. Furthermore, some studies have shown that interventions targeting importin-related pathways may also influence the host adaptability of different influenza virus subtypes [83].

**Figure 4:**
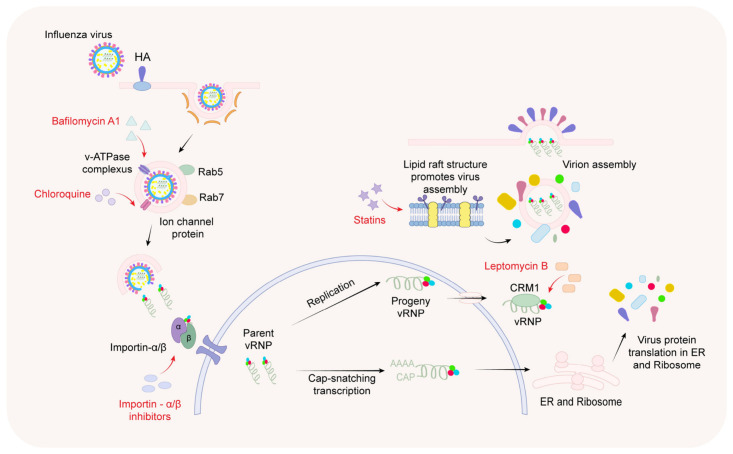
Targeting key host pathways for influenza virus inhibition.

In addition to nuclear transport, host lipid metabolism and membrane homeostasis are closely associated with viral replication. Influenza virus budding depends on lipid raft structures enriched in cholesterol and sphingolipids [84]; therefore, modulation of cholesterol metabolism may affect viral particle assembly and release. For example, statins have been proposed to possess potential antiviral activity because they influence cholesterol biosynthesis pathways [85]. However, current evidence is derived primarily from experimental studies and observational analyses, whereas clinical and translational findings remain inconsistent. Furthermore, host signaling pathways such as PI3K–Akt and MAPK also play regulatory roles in viral replication, and inhibitors targeting these pathways have demonstrated anti-influenza activity in several studies [62,86] (Figure 4).

In addition, many host pathways exhibit relatively narrow therapeutic windows [87], as partial inhibition may be insufficient to suppress viral replication, whereas excessive inhibition may impair essential cellular functions. Identifying intervention levels that maintain antiviral efficacy while preserving host homeostasis remains a critical challenge in the development of host-directed antiviral therapies.

Finally, due to the high conservation of processes such as nuclear transport, endosomal trafficking, and lipid metabolism across humans, pigs, and birds, interventions targeting these host pathways may have potential applications in both human and livestock influenza control. Overall, targeting key host pathways offers considerable antiviral potential because many viruses rely on similar cellular processes. However, because these pathways also contribute to normal cellular functions, safety and selectivity remain important considerations in therapeutic development.

This schematic highlights pharmacological inhibitors (red boxes) that block host factors critical for influenza virus replication. Bafilomycin A1 and Chloroquine target endosomal acidification (v-ATPase) and trafficking (Rab5/Rab7), respectively. Statins disrupt lipid raft structures required for virion assembly, while Leptomycin B inhibits CRM1-mediated nuclear export of progeny vRNPs. Additional interventions include blockade of Importin-α/β-dependent nuclear import, cap-snatching transcription, and viral protein translation at the ER/ribosome. These host-directed strategies collectively suppress multiple stages of the viral life cycle.

### 5.2. Immune Strategy of Host Targeted Intervention

In addition to directly interfering with viral replication, modulation of the host immune response represents another important direction in host-targeted antiviral research. The innate immune system, particularly the interferon (IFN) pathway, plays a critical role in restricting early influenza virus replication. Upon recognition of viral RNA by pattern recognition receptors such as RIG-I, type I and type III interferons are induced, which subsequently activate a wide range of interferon-stimulated genes (ISGs), thereby inhibiting viral replication and spread [27,88].

Consequently, exogenous administration of interferons or enhancement of related signaling pathways can strengthen the host antiviral response during the early stages of infection. Existing studies have demonstrated that IFN-α and IFN-β exert inhibitory effects against various influenza viruses [89], reducing viral replication and alleviating disease severity. In addition, immunostimulants such as poly(I:C), which activate the RIG-I/MDA5 signaling pathway [90], have been used to enhance innate antiviral immunity. Certain small-molecule compounds can also promote an antiviral cellular state by enhancing interferon signaling and increasing the expression of ISGs [91].

However, excessive activation of the immune response may also result in severe tissue damage. During infection with highly pathogenic influenza viruses, dysregulated inflammatory responses characterized by excessive production of cytokines and chemokines can contribute to lung injury and acute respiratory distress syndrome (ARDS) [92]. Therefore, a major objective of immunomodulatory strategies is to suppress excessive inflammation while preserving effective antiviral responses. For example, glucocorticoids, JAK inhibitors, and certain anti-inflammatory cytokine modulators have been investigated as potential approaches to mitigate severe inflammatory responses associated with influenza infection [93]. However, the use of glucocorticoids remains controversial because excessive suppression of antiviral immunity may delay viral clearance and increase the risk of secondary infections [94]. Additionally, modulation of inflammatory mediators such as IL-6 has been proposed as a strategy to reduce influenza-associated immunopathology, although its clinical efficacy requires further evaluation [95].

Overall, the primary goal of immunomodulatory strategies is to maintain a balance between antiviral immunity and inflammation control. Particularly in cases of severe influenza or highly pathogenic avian influenza infection, appropriate regulation of the host immune response may not only help limit viral replication but also reduce tissue damage and improve disease outcomes. Furthermore, in large-scale livestock production systems, enhancing innate immune responses to reduce viral shedding and transmission risk may also have practical value.

### 5.3. Small Molecules and Biologics

In host-targeted antiviral research, small-molecule compounds and biologics are the most common forms of intervention. Unlike traditional antiviral drugs that directly target viral proteins, these approaches primarily inhibit viral replication indirectly by modulating key host cellular pathways or immune responses. Small-molecule inhibitors typically target critical host components, such as kinases, transporters, and metabolic enzymes. For example, the PI3K–Akt and MAPK signaling pathways play important roles in influenza virus replication, and several kinase inhibitors have been shown to reduce viral replication levels [62]. Verdinexor, an inhibitor of the nuclear export protein CRM1/exportin 1, interferes with the nuclear export of viral vRNP complexes, thereby suppressing viral replication [96]. In addition, certain compounds that affect lipid metabolism, such as statins, have been proposed to influence viral assembly and budding by regulating cholesterol metabolism and lipid raft organization [24,85]. These small-molecule agents generally benefit from well-established development pipelines, flexible routes of administration, and relatively straightforward large-scale manufacturing.

Biologics, including monoclonal antibodies, recombinant cytokines, and fusion proteins, are also widely used in host-targeted antiviral research. For example, recombinant IFN-α and IFN-β can enhance innate immune responses and improve the antiviral capacity of host cells [89], whereas antibodies targeting inflammatory mediators such as IL-6 have been investigated for reducing severe influenza-associated inflammatory damage [95]. In addition, certain biologics targeting host receptors or immunomodulatory molecules have been used to intervene in the course of viral infection or to correct immune dysregulation [97].

Compared with small-molecule drugs, biologics generally exhibit greater target specificity. However, their production costs, storage requirements, and modes of administration may limit their applicability in certain settings. Finally, host-factor dependency may vary substantially among tissues and cell types. A target that exhibits strong antiviral effects in respiratory epithelial cells may have different biological consequences in immune, hepatic, or intestinal tissues, thereby complicating efficacy and safety assessments. Although host-directed antiviral strategies offer advantages in reducing the likelihood of antiviral resistance and potentially broadening antiviral activity, their practical application still requires careful evaluation of safety, efficacy, and translational feasibility.

## 6. Host-Targeted and Cross-Species Antiviral Strategies in Livestock

Livestock serve as critical ecological niches for the natural circulation and interspecies transmission of influenza viruses. In particular, high-density swine and poultry production systems provide favorable conditions for sustained viral transmission and genetic reassortment. This not only complicates disease control at the animal level but also increases the risk of the emergence and cross-species transmission of novel influenza viruses to humans. Therefore, the development of effective, scalable antiviral strategies for livestock populations is of considerable importance for both public health security and the sustainability of the livestock industry. Building upon the concept of host-targeted antiviral approaches, increasing attention has been directed toward the identification of conserved host mechanisms shared among humans, swine, and poultry. Such targets may facilitate the development of cross-subtype antiviral strategies against diverse influenza virus strains and, in some cases, may offer broader applicability across multiple host species.

### 6.1. Influenza Virus Prevalence and Risks in Livestock

In livestock populations such as pigs and poultry, influenza viruses often circulate persistently over extended periods and are prone to genetic reassortment among different subtypes. For example, multiple influenza virus subtypes originating from human, avian, and swine populations can co-circulate within pig herds [98]. Pigs have traditionally been regarded as “mixing vessels” because their respiratory tracts express both α2,3-linked and α2,6-linked sialic acid receptors [99], which may facilitate infection by viruses originating from different host species. However, the emergence of reassortant viruses is influenced not only by receptor distribution but also by viral introductions from human and avian populations, farm management practices, animal movement, surveillance intensity, and regional ecological conditions [100].

The emergence of the 2009 H1N1 pandemic virus is widely believed to have involved multiple reassortment events among swine influenza lineages [101]. Nevertheless, subsequent studies have emphasized that reassortment dynamics are shaped by complex interactions among host biology, viral ecology, animal production systems, and geographic factors. Moreover, recent surveillance studies suggest that the role of pigs in influenza evolution should be considered within a broader One Health framework [102]. Reassortment opportunities depend not only on the biological susceptibility of pigs but also on the frequency of virus exchange among humans, livestock, poultry, and wildlife. Consequently, the epidemiological significance of pigs as mixing hosts may vary across production systems and geographical regions. In poultry production systems, outbreaks of highly pathogenic avian influenza viruses, such as H5N1 and H5N8, often result in substantial economic losses and high mortality rates. For example, outbreaks of H5 subtype avian influenza in certain regions frequently require large-scale culling to control transmission, thereby causing major disruptions to the poultry industry. In addition, waterfowl, including ducks and geese, serve as natural reservoirs of influenza viruses and play an important role in maintaining long-term viral circulation [103].

**Figure 5:**
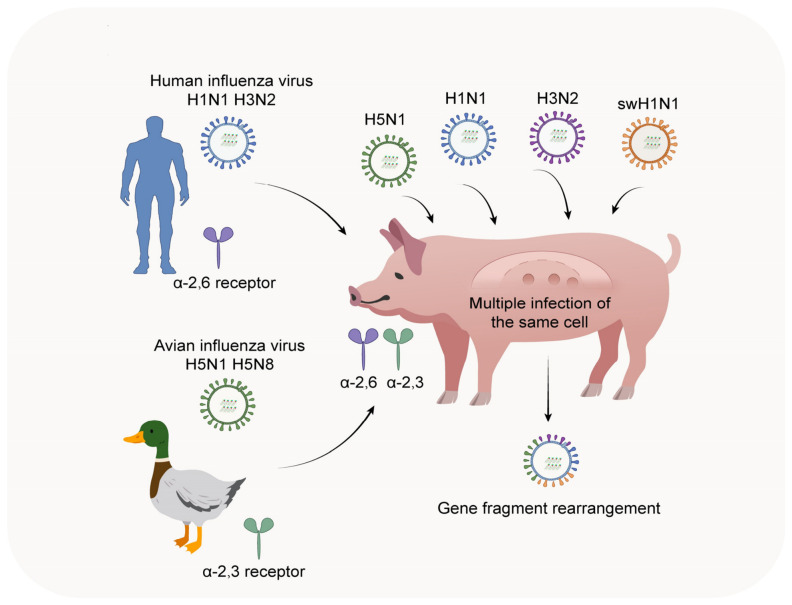
Role of pigs in influenza A virus reassortment at the human–avian interface.

At present, influenza prevention and control in livestock populations continue to rely primarily on vaccination and biosecurity measures [104], including farm isolation, personnel and equipment disinfection, and transportation controls. However, because influenza viruses undergo frequent antigenic variation—particularly continuous antigenic drift in the HA and NA proteins—vaccine effectiveness often varies across geographical regions and epidemic seasons. For instance, avian influenza vaccines may provide only limited protection against newly emerging reassortant strains [105], thereby reducing overall disease-control effectiveness (Figure 5).

On the other hand, individualized treatment strategies are difficult to implement on a large scale in intensive livestock production systems, necessitating a greater reliance on population-level prevention and control measures. This creates an increasing need for antiviral approaches capable of providing activity across multiple influenza virus subtypes and, where feasible, applicability across different host species. Therefore, the development of robust antiviral strategies that are effective against diverse influenza virus subtypes and suitable for large-scale livestock production systems has considerable practical significance and translational potential.

Pigs express both α-2,6 (human-type) and α-2,3 (avian-type) sialic acid receptors on respiratory epithelial cells, enabling co-infection of human (e.g., H1N1, H3N2, swH1N1) and avian (e.g., H5N1, H5N8) influenza viruses within the same cell. This dual receptor tropism facilitates genetic reassortment among different viral strains, generating novel progeny viruses with pandemic potential.

### 6.2. Cross-Species Conserved Host Targets

The identification of host factors that are conserved across multiple host species and play essential roles in viral replication represents a key objective of host-targeted antiviral strategies. Such factors are typically involved in fundamental cellular processes, including membrane trafficking, nucleocytoplasmic transport, and innate immune signaling, and therefore often exhibit substantial functional conservation among humans, swine, and poultry.

For example, the importin-α/β nuclear transport system mediates the nuclear import of influenza viral ribonucleoprotein complexes (vRNPs) in a wide range of host cells [106]. Similarly, the ANP32 protein family represents one of the best-characterized host factor families that combines evolutionary conservation with species-specific functional variation [20]. Although ANP32 proteins are widely conserved among vertebrates, their capacity to support influenza virus polymerase activity differs markedly among host species. Avian ANP32A contains a unique 33-amino-acid insertion that is absent in mammalian ANP32 proteins [107]. Functional complementation studies have demonstrated that expression of avian ANP32A in mammalian cells can restore the activity of avian influenza polymerases, highlighting the critical role of species-specific ANP32 variation in determining host range and cross-species adaptation [20,21]. These differences contribute to host restriction barriers and influence the ability of avian influenza viruses to establish infection in mammalian hosts. Consequently, ANP32 proteins provide an important model for understanding how conserved host factors can simultaneously influence viral replication and cross-species transmission dynamics. In addition, CRISPR-based screening studies have consistently identified ANP32A and ANP32B as key host factors required for influenza virus polymerase activity [108]. Other host pathways, including the Rab5/Rab7-mediated endosomal trafficking axis and the JAK–STAT signaling pathway [15,109], are also highly conserved across multiple host species and play central regulatory roles during influenza virus infection. Evidence supporting the conservation of these pathways is available at multiple levels. Comparative sequence analyses have shown that key components of the nuclear transport machinery, Rab GTPases, and JAK–STAT signaling proteins exhibit high amino acid sequence conservation among humans, swine, and avian species. Transcriptomic studies further indicate that many of these genes are constitutively expressed in respiratory and immune-related tissues across different hosts, although their expression levels may vary according to tissue type and infection status. Importantly, functional conservation does not necessarily imply complete biological equivalence. Cross-species complementation experiments have demonstrated that avian ANP32A efficiently supports avian influenza polymerase activity [20], whereas mammalian ANP32 proteins often require adaptive mutations within the viral polymerase complex to achieve comparable levels of activity. These findings suggest that the evaluation of conserved host factors should extend beyond sequence similarity and include assessments of functional compatibility and species-specific regulatory contexts.

Comparative analyses integrating cross-species multi-omics datasets, including transcriptomic profiles and CRISPR screening results from human, swine, and avian cells, can further identify core pathways involved in viral replication across different hosts. Such conserved host pathways may serve as candidate targets for the development of antiviral strategies with activity against multiple influenza virus subtypes and, potentially, other RNA viruses. However, because many of these targets participate in essential cellular processes, their therapeutic exploitation requires careful evaluation of safety, tissue specificity, and species-dependent biological functions.

### 6.3. Types of Broad-Spectrum Antiviral Strategies

Broad-spectrum antiviral strategies currently being explored in livestock systems can be broadly divided into three categories. The first category involves targeting host pathways that are commonly exploited by viruses, with the aim of inhibiting multiple viruses by disrupting key cellular processes required for viral replication. This approach has been investigated most extensively, particularly in pathways associated with nuclear transport, endocytic trafficking, and cellular metabolism, where substantial experimental evidence has accumulated. Theoretically, such strategies offer considerable potential for inhibiting multiple influenza virus subtypes and, in some cases, other viruses that rely on similar host mechanisms. However, they may also carry a higher risk of adverse effects because many of the targeted pathways are essential for normal cellular functions. For example, interventions targeting endocytosis and endosomal acidification, such as those affecting v-ATPase-associated pathways [110], can impair the entry of multiple influenza virus subtypes. Similarly, CRM1/exportin 1, a key component of the nuclear export pathway, has been implicated in the replication of various RNA viruses, and its inhibition may produce broad antiviral activity [96].

The second category focuses on modulation of the host’s innate immune response. By enhancing interferon signaling or the activity of downstream effector molecules, these strategies aim to strengthen the host’s overall antiviral capacity. For example, exogenous interferons, such as IFN-α, can induce the expression of antiviral genes, including ISG15, OAS1, and MX1, in both avian and porcine cells, thereby limiting viral replication during the early stages of infection [111]. In addition, molecules capable of activating RIG-I-like receptor signaling have been investigated as a means of enhancing host defenses against multiple influenza virus subtypes [112]. Compared with direct interference in fundamental cellular processes, immune-modulatory approaches may provide broader antiviral coverage while preserving essential cellular functions. However, their efficacy is often influenced by host-specific factors, the stage of infection, and viral virulence. Variability in immune responses among animal populations may also contribute to inconsistent outcomes.

The third category involves host metabolic regulation, which seeks to impair the cellular environment required for viral replication by modulating lipid metabolism, energy metabolism, or related pathways. For example, influenza virus replication and budding depend on cholesterol-rich lipid raft structures [24]; therefore, strategies that alter cholesterol biosynthesis or membrane lipid composition, including those investigated using statins, may influence viral particle assembly. Furthermore, glycolytic and mitochondrial metabolic pathways are frequently reprogrammed during viral infection [113], and interventions targeting these pathways have demonstrated potential antiviral effects. Compared with strategies targeting nuclear transport or immune signaling, metabolic interventions may have a more favorable safety profile because they generally exert indirect effects on viral replication. Nevertheless, their antiviral efficacy is often modest, and metabolic regulation can differ substantially among host species. Consequently, further investigation is required to evaluate their translational potential in livestock systems (Table 3).

Overall, these approaches share a common principle: rather than directly targeting viral proteins, they exploit relatively stable host biological processes that are less susceptible to rapid viral mutation. As a result, host-directed strategies may offer advantages over direct-acting antivirals in reducing the likelihood of resistance development and targeting conserved host mechanisms. However, their efficacy across different influenza virus subtypes, safety profiles, and practical applicability in livestock production systems remains to be fully validated. Food safety considerations are particularly important in livestock production settings. In addition to drug residue concerns, factors such as withdrawal periods, regulatory approval requirements, administration feasibility, and potential effects on product quality must be carefully evaluated before large-scale implementation of host-targeted antiviral strategies.

### 6.4. Broad-Spectrum Antiviral Strategy from a One Health Perspective

Within the One Health framework, influenza prevention and control should not be confined to a single host system but should instead consider the interconnected transmission network among humans, livestock, and poultry. In particular, the cross-species transmission chain involving wild waterfowl, poultry, pigs, and humans represents an important ecological foundation for the emergence and dissemination of novel influenza viruses [42,114]. Influenza outbreaks in livestock not only result in reduced productivity, increased mortality, and substantial economic losses but may also create opportunities for the emergence of novel influenza viruses with zoonotic potential [5]. Therefore, reducing viral replication and transmission in pigs and poultry is not only an animal health objective but also an important public health measure for minimizing the risk of human exposure.

The identification of conserved host targets provides new opportunities to support this goal. Many host-dependent pathways, including the nuclear transport system, endocytic and endosomal trafficking pathways, interferon signaling, and lipid metabolism, exhibit a substantial degree of conservation among humans, pigs, and poultry [41]. Targeting these pathways may provide a foundation for the development of antiviral strategies applicable to both human and veterinary medicine. For example, interventions targeting CRM1/exportin 1, the JAK–STAT signaling pathway, or cholesterol metabolism have demonstrated antiviral potential in multiple host systems [96].

However, significant differences in immune responses, metabolic characteristics, drug metabolism, and treatment tolerance exist among different host species, making it difficult to directly apply a single intervention strategy across all hosts [41]. Consequently, the development of antiviral approaches with activity across multiple host species requires a careful balance between conserved host mechanisms and species-specific biological differences [115]. On the one hand, priority should be given to identifying host factors that are both highly conserved across species and essential for viral replication. On the other hand, intervention intensity, administration routes, safety assessment, and practical implementation should be optimized according to the physiological characteristics of individual host species.

An important direction for future research is the establishment of cross-species multi-omics integration frameworks. By jointly analyzing transcriptomic, proteomic, interactomic, and CRISPR screening datasets derived from humans, pigs, poultry, and wild birds, it will be possible to identify regulatory networks that play central roles across different host species more systematically. At the same time, the application of artificial intelligence to multi-source data integration, target prioritization, and network analysis is expected to further improve the efficiency and accuracy of host-target discovery. Ultimately, integrated human–animal surveillance and control systems developed under the One Health framework may become an important component of future strategies for the prevention and control of influenza and other emerging RNA viruses.

## 7. Challenges and Limitations

Although advancements in multi-omics technologies and artificial intelligence methods have significantly propelled progress in host-targeted antiviral research and provided new insights for broad-spectrum antiviral strategies, the transition from basic research to practical application still faces multiple constraints. These issues involve both the biological complexity of host–virus interactions and technical and practical barriers in data analysis and translation.

### 7.1. Biological and Safety Challenges of Host-Targeted Interventions

A major challenge for host-targeted antiviral strategies arises from the multifunctional nature of host factors. Many host proteins exploited or regulated by viruses are also involved in essential cellular processes, including nuclear transport, protein translation, and metabolic regulation. Consequently, interventions targeting these proteins may disrupt normal cellular homeostasis and lead to toxicity or unintended physiological effects.

In addition, functional redundancy and compensatory mechanisms are common within host cells. When a particular pathway is inhibited, alternative pathways may partially compensate for its function, allowing essential cellular processes to continue [116]. As a result, inhibition of a single host factor may not always produce sustained or robust antiviral effects in vivo. Another challenge is the context-dependent nature of host-factor function. Different cell types and tissue environments often exhibit distinct levels of dependence on the same host pathway. For example, epithelial cells and immune cells may respond differently to the modulation of interferon signaling or metabolic pathways [117]. Consequently, observations obtained from immortalized cell lines do not always accurately predict outcomes in primary tissues or animal infection models.

Furthermore, many host pathways have relatively narrow therapeutic windows. Partial inhibition may be insufficient to suppress viral replication effectively, whereas excessive inhibition can impair normal cellular functions and increase the risk of adverse effects. These challenges are particularly relevant for highly conserved pathways, such as nuclear transport, endosomal trafficking, and cellular metabolism, which are critical for both viral replication and host physiology. Collectively, these factors complicate host-target validation and highlight the need for careful evaluation of efficacy, safety, tissue specificity, and therapeutic windows during the development of host-directed antiviral interventions.

### 7.2. Challenges in Multi-Omics Integration and AI-Assisted Target Prioritization

The heterogeneity of multi-omics data represents one of the most significant technical bottlenecks in current host-targeted antiviral research. Different omics platforms, including transcriptomics, proteomics, and interactomics, differ substantially in data structure, detection sensitivity, and dynamic range, making cross-omics standardization and integration challenging. For example, changes in mRNA abundance do not always correlate with protein expression levels, while post-translational modifications add further complexity to biological interpretation [118].

In addition, influenza virus infection is a highly dynamic process, with host responses varying considerably across different stages of infection, including viral entry, replication, and release. However, many studies still rely on single-time-point or endpoint sampling, limiting the ability to capture temporal regulatory dynamics. This static view may obscure stage-specific host dependencies and reduce the biological relevance of computational predictions. Publicly available influenza datasets are also frequently biased toward particular viral strains, cell lines, and experimental systems. Such sampling biases can affect model generalizability and lead to the overrepresentation of specific pathways or host factors. Moreover, many AI models are trained predominantly on data generated from a limited number of cell lines, such as A549, 293T, and MDCK cells, which may not accurately reflect host-factor dependencies in primary tissues or in vivo infection settings [119].

Another limitation is the lack of well-defined negative datasets. Most studies focus on identifying host factors that promote or restrict viral replication, whereas experimentally validated non-associated genes are rarely reported. This imbalance can introduce bias during model training and reduce prediction reliability. Potential information leakage between training and testing datasets represents an additional concern. For example, related genes, pathways, or interaction networks may appear in both datasets, resulting in overly optimistic estimates of model performance. Importantly, examples from influenza host-factor research illustrate how dataset heterogeneity can affect reproducibility. Several genome-wide RNAi screens conducted in different cell lines and experimental systems identified hundreds of candidate host factors required for influenza virus replication; however, the overlap among the reported gene sets was unexpectedly low. In some cases, host factors identified as important in one study could not be reproduced in subsequent screens performed using different viral strains or cellular backgrounds. These inconsistencies were attributed to variations in experimental design, screening methodology, statistical thresholds, and data-processing pipelines. Such findings highlight the risk that AI models trained on heterogeneous datasets may prioritize context-specific or false-positive candidates rather than universally relevant host factors. Without rigorous validation, AI-assisted target prioritization may inadvertently amplify existing biases and generate predictions that are difficult to reproduce across independent studies.

Furthermore, many published AI models are evaluated only on the datasets used for model development and lack validation using independent external datasets. Consequently, their applicability across different host species, viral strains, and experimental conditions remains uncertain. Therefore, future studies should emphasize standardized multi-omics data generation, cross-study harmonization, independent external validation, and experimental verification of computational predictions. Integrating AI-assisted target discovery with functional genomics, molecular virology, and animal infection studies will be essential for translating computationally identified host factors into practical antiviral targets for both human and veterinary applications.

### 7.3. Translational Challenges in Human and Livestock Applications

In the transition from basic research to practical application, host-targeted antiviral strategies still face multiple practical constraints. First, safety remains the primary consideration, particularly for host targets involved in fundamental cellular processes, such as nuclear transport systems and core metabolic pathways. Long-term or systemic intervention in these pathways may lead to unintended side effects, including immune dysfunction and metabolic disturbances.

Second, in practical settings such as livestock production systems, factors including production costs, administration methods, and the feasibility of large-scale implementation must be carefully evaluated. For example, the sustained use of immunomodulators or metabolic intervention agents in intensive farming systems may be constrained by economic costs and operational complexity.

In addition, food safety, drug residues, and withdrawal-period requirements represent important considerations for the application of host-targeted interventions in food-producing animals. Candidate compounds must not only demonstrate antiviral efficacy but also comply with regulatory standards governing residue depletion in edible tissues and animal-derived products. Prolonged withdrawal periods may reduce production efficiency, increase management costs, and limit large-scale adoption in commercial livestock systems. Therefore, residue profiles, pharmacokinetics, and withdrawal-period assessments should be incorporated early in the development of host-targeted antiviral strategies for livestock. Beyond antiviral efficacy, practical evaluation frameworks should also be integrated into the early stages of candidate development. For livestock applications, key assessment criteria may include antiviral effectiveness, cost-effectiveness, ease of administration, withdrawal-period requirements, residue risks in edible products, animal welfare considerations, and compatibility with existing farm management practices. For example, antiviral strategies that require repeated individual administration may be difficult to implement in large-scale poultry or swine production systems, whereas approaches compatible with feed- or water-based delivery may offer greater scalability. Similarly, candidate interventions associated with prolonged withdrawal periods or complex residue-monitoring requirements may face substantial regulatory and economic barriers despite demonstrating antiviral activity under experimental conditions. Therefore, translational feasibility should be evaluated alongside biological efficacy throughout the processes of target validation and drug development.

Furthermore, substantial differences exist among species with respect to immune system architecture, metabolic characteristics, drug metabolism, and susceptibility to viral infection, making it difficult to directly translate host-targeted strategies validated in one species to another. For instance, intervention strategies that are effective in porcine cell systems may produce different outcomes in avian or human systems. Collectively, these factors continue to limit the translation of host-targeted antiviral strategies from experimental research into practical applications.

## 8. Conclusions and Future Perspectives

Influenza virus replication depends on a diverse range of host factors that regulate viral entry, genome replication, intracellular trafficking, and innate immune responses. Among the host targets identified to date, pathways involved in nuclear transport, ANP32-mediated polymerase support, endosomal trafficking, and interferon signaling have been consistently implicated in influenza virus replication and therefore remain important targets for further investigation because of their central biological roles and potential conservation across host species. However, the biological importance of many host factors also presents challenges related to safety, tissue specificity, and therapeutic windows, underscoring the need for careful target prioritization. Multi-omics technologies have greatly expanded the capacity to identify host factors by integrating evidence from functional genomics, transcriptomics, proteomics, and interactomics. Importantly, combining these complementary datasets with AI-assisted prioritization frameworks provides a practical approach for distinguishing biologically relevant and potentially druggable targets from large candidate pools. Nevertheless, challenges related to data heterogeneity, model interpretability, external validation, and experimental verification remain major barriers to translation.

From a One Health perspective, the identification of conserved host pathways across humans, swine, poultry, and other animal hosts represents an important opportunity for the development of host-directed antiviral strategies with cross-subtype influenza control potential. Over the next three to five years, progress in this field will likely depend on four key developments: (i) the generation of standardized multi-species multi-omics resources; (ii) the application of explainable AI methods for host-target prioritization; (iii) systematic experimental validation using functional genomics, molecular virology, and animal infection models; and (iv) the establishment of translational evaluation frameworks that incorporate efficacy, safety, scalability, and regulatory considerations. Collectively, these advances will accelerate the discovery of conserved host targets and support the development of next-generation broad-spectrum antiviral strategies for both human and veterinary medicine within a One Health framework.

Subsequently, candidate targets should undergo systematic validation through CRISPR-based functional screening, mechanistic studies, and animal infection models, followed by comprehensive evaluation of safety, efficacy, scalability, and regulatory feasibility. For livestock applications, additional factors—including administration routes, residue risks, withdrawal periods, and cost-effectiveness—should be incorporated early in the development process. Ultimately, the integration of comparative multi-omics, artificial intelligence, and experimental validation within a standardized translational framework provides a practical roadmap for accelerating the development of next-generation host-directed antiviral strategies against influenza and other emerging RNA viruses.

## Figures and Tables

**Table 1:** Representative host factors and their translational potential for host-directed antiviral development.

Host Factor	Role	Validation Status	Cross-Species Conservation	Therapeutic Potential
Importin-α/β	Nuclear import	Cell + animal studies	High	High
ANP32A/B	Polymerase support	Cell + animal studies	High	High
CRM1/XPO1	Nuclear export	Small-molecule inhibitors available	High	High
IFITM3	Restriction factor	Genetic association studies	Moderate	Moderate
MxA/MX1	Restriction factor	Strong mechanistic evidence	Moderate	Moderate
PKR	Translation inhibition	Extensive antiviral evidence	High	Limited (toxicity concerns)

**Table 2:** Examples of influenza host factors identified by functional genomics studies.

Host Factor	Function	Evidence	Druggability	Potential Limitations
SLC35A1	CMP-sialic acid transporter	CRISPR KO	Low	Essential for host glycosylation
CMAS	Sialic acid synthesis	CRISPR KO	Low	High toxicity risk
ANP32A	Polymerase cofactor	CRISPR KO+ mechanistic studies	Moderate	Host cellular functions unclear
ANP32B	Polymerase cofactor	CRISPR KO+ mechanistic studies	Moderate	Functional redundancy

**Table 3:** Representative host-directed broad-spectrum antiviral strategies for influenza control in the livestock system.

Candidate Strategy	Target Pathway	Species Investigated	Evidence Level	Delivery Route	Residue/Safety Concerns	Scalability in Livestock
v-ATPase inhibitors (e.g., Bafilomycin A1)	Endosomal acidification/viral entry	Human cell lines, limited animal studies	Mechanistic proof of concept	Experimental only	High cellular toxicity; unsuitable for food animals	Very low
CRM1/XPO1 inhibitors (e.g., Verdinexor)	Nuclear export of vRNP complexes	Human, swine cell models	In vitro and limited in vivo studies	Oral or injectable (experimental)	Potential toxicity due to interference with host nuclear transport	Low
Interferon-α (IFN-α)	JAK–STAT signaling, ISG induction	Swine, poultry, humans	In vitro and animal studies	Injection, mucosal delivery, aerosol	Risk of excessive immune activation; cost considerations	Moderate
RIG-I/MDA5 agonists (e.g., poly(I:C))	Innate immune activation	Poultry, swine, experimental mammalian models	Experimental studies	Injection, aerosol, adjuvant formulations	Excessive inflammation and reduced productivity possible	Moderate
JAK inhibitors	Cytokine signaling regulation	Mainly human studies; limited veterinary data	Preclinical and clinical evidence (human)	Oral administration	Immunosuppression and secondary infection risks	Low–moderate
Cholesterol metabolism modulators (e.g., statins)	Lipid raft formation and viral budding	Humans, experimental animal models	Mixed experimental evidence	Oral administration/feed supplementation	Uncertain antiviral efficacy; residue evaluation required	Moderate
Glycolysis inhibitors	Cellular energy metabolism	Cell culture and experimental animal studies	Early-stage experimental evidence	Oral administration (potential)	May affect growth performance and feed conversion efficiency	Low
Mitochondrial metabolism modulators	Cellular bioenergetics and antiviral responses	Experimental models	Early-stage experimental evidence	Oral administration (potential)	Potential impact on host metabolism and productivity	Low
Host-targeted RNA therapeutics (siRNA/CRISPR-based approaches)	Host dependency factors (e.g., ANP32, importin pathways)	Mainly laboratory models	Proof of concept	Nanoparticle delivery, viral vectors	Delivery efficiency, biosafety, regulatory concerns	Currently low
Multi-target immunomodulatory strategies	Interferon and innate immune pathways	Swine and poultry experimental studies	Preclinical evidence	Feed additives, aerosol, vaccination-associated delivery	Immune imbalance and variable responses among species	High potential

## Data Availability

No new data were created or analyzed in this study. Data sharing is not applicable to this article.
